# Cellular Signaling and Potential New Treatment Targets in Diabetic Retinopathy

**DOI:** 10.1155/2007/31867

**Published:** 2007-12-17

**Authors:** Zia A. Khan, Subrata Chakrabarti

**Affiliations:** Department of Pathology, University of Western Ontario, London, Ontario, Canada N6A 5C1

## Abstract

Dysfunction and death of microvascular cells and imbalance between the production and the degradation of extracellular matrix (ECM) proteins are a characteristic feature of diabetic retinopathy (DR). Glucose-induced biochemical alterations in the vascular endothelial cells may activate a cascade of signaling pathways leading to increased production of ECM proteins and cellular dysfunction/death. Chronic diabetes leads to the activation of a number of signaling proteins including protein kinase C, protein kinase B, and mitogen-activated protein kinases. These signaling cascades are activated in response to hyperglycemia-induced oxidative stress, polyol pathway, and advanced glycation end product formation among others. The aberrant signaling pathways ultimately lead to activation of transcription factors such as nuclear factor-κB and activating protein-1. The activity of these transcription factors is also regulated by epigenetic mechanisms through transcriptional coactivator p300. These complex signaling pathways may be involved in glucose-induced alterations of endothelial cell phenotype leading to the production of increased ECM proteins and vasoactive effector molecules causing functional and structural changes in the microvasculature. Understanding of such mechanistic pathways will help to develop future adjuvant therapies for diabetic retinopathy.

## 1. INTRODUCTION

Diabetic retinopathy (DR), the most common cause of blindness in the working population, is also the most frequent manifestation of the diabetic microvascular disease [1, 2]. Nearly all people with type 1 and more than half with type 2 diabetes develop retinopathy [3]. Retinal complications in chronic diabetes may culminate from microvascular dysfunction, neuroglial abnormalities, and metabolic imbalance including the lack (type 1) or excess (type 2) of insulin. Microvascular alterations comprise of microvascular endothelial cell (EC) dysfunction, vessel leakage, and occlusions. Clinically, DR can be classified as nonproliferative DR (NPDR) and proliferative DR (PDR) [3–7]. NPDR is structurally characterized by capillary basement membrane (BM) thickening, pericyte loss, microaneurysms, increased permeability, exudate deposits, and retinal microinfarcts [3–7]. Diabetic macular edema may lead to significant visual impairment in NPDR. Also NPDR has further been divided in progressive stages: mild, moderate and severe. The later stages (sometimes also called preproliferative retinopathy) show increased retinal damage as evidenced by increased retinal vascular blockage and infarcts. If left untreated, proliferative retinopathy characterized by abnormal neovascularization in the retina develops. The devastating outcome of the proliferative retinopathy is hemorrhage and tractional retinal detachment.

Although it has been customary to consider a common origin of microvascular disease in both type 1 and type 2 diabetes, recent evidence suggests considerable heterogeneity in the development and progression among the two populations. Clinical studies reveal that vision threatening retinopathy is usually because of neovascularization in type 1 and maculopathy in type 2 patients [8, 9]. Clinical trials have shown that good glycemic control can reduce the development of retinopathy in both type 1 [10] and type 2 [11] diabetic patients. Current treatment for PDR and maculopathy is carried out by laser photocoagulation which prevents further loss of vision. However, these therapeutic modalities are ineffective against restoring diminished visual acuity. Understanding the molecular basis of DR pathogenesis may offer better therapeutic regimens. Towards this aim, investigators have studied the effects of high glucose levels in animal models of the disease and cultured cells. These in vivo and in vitro models do not fully recapitulate the disease process observed in humans. The proliferative stage of the DR has not been observed in the well studied rodent models. However, both the in vitro and the in vivo models do offer insight into the early *molecular* changes which may dictate the more advanced stages of the disease. This review will highlight some of these findings in both cultured vascular ECs and animal models of diabetes with an emphasis on cell signaling and the early molecular changes mediating microvascular alterations in DR.

## 2. STRUCTURAL AND FUNCTIONAL CHANGES IN DR

### 2.1. Alteration of vasoactive effector molecules

Vasoactive effector molecules play important roles in both early and late stages of DR. Hyperglycemia leads to increased vasoconstriction and impaired endothelium-dependent vasodilation [12–20]. Increased expression of endothelin-1 (ET-1), the most potent vasoconstrictor, has been shown in diabetes (reviewed in [13]). Increased ET-1 mediates vasoconstriction in the retina which is readily prevented by inhibiting ET receptor signaling [19]. In cultured cells, we have also shown that ET-1 increases EC permeability and extracellular matrix (ECM) protein expression [13, 21–23] possibly through protein kinase C (PKC) activation [24].

Endothelium-dependent vasodilatory responses have been shown to be dampened in diabetics [14–18]. Data accumulated to date indicates increased levels of endothelial (e-) and inducible (i-) nitric oxide synthase (NOS) enzymes in response to high levels of glucose [25, 26]. Such a phenomenon is also evident in animal and human diabetes (reviewed in [27]). In addition, a number of molecular pathways which are altered in diabetes may increase the activity of NOS enzymes; these include vascular endothelial growth factor (VEGF) [28], and PKC and protein kinase B signaling pathways [28–30]. A few theories can be formulated to account for the discrepancy between increased NOS expression/activity, NO levels and impaired vasodilation. Increased NO production but decreased bioavailability may be one explanation. Secondly, ET-induced vasoconstriction may overwhelm the vasodilatory action of NO. In the context of diabetic milieu (increased ET levels and oxidative stress), NO may also mediate other cellular activities such as EC proliferation as suggested by the increased NO level observed during the proliferative phase of DR. Finally, NO function may be influenced by heme oxygenase-1 (HO-1) [25]. We have shown that diabetes causes oxidative DNA damage in several tissues including the retina and alter blood flow through the induction of HO-1 [25, 27, 31]. HO-1 agonist in ECs and animals mimics the effects of high glucose and diabetes by increasing NOS levels.

An extensive interaction exists among these vasoactive effector molecules which determine the expression/production and function of the respective molecule [13, 26]. Taken together, these studies have shown that alteration of these vasoactive effector molecules may be instrumental in the production of early changes in DR such as blood flow alterations, increased permeability, and ECM deposition, eventually leading to nonperfusion of the retinal tissue.

### 2.2. Cellular dysfunction and death—effect on retinal vascular function

One early event in DR is the loss of pericytes from capillaries and venules [32, 33]. Pericyte loss is believed to contribute to the earliest clinically visible manifestation of DR, microaneurysms [34]. This loss may occur due to increased oxidative stress, toxicity from high levels of glucose, and other factors. Recently, autoantibodies against pericytes have been detected in the sera of diabetic patients [35, 36]. Loss of pericytes may not only affect the vascular tone but may also lead to EC phenotypical changes. The integral communication between ECs and contractile cells of the vascular wall is increasing being realized (for review, see [37–39]). In addition to the indirect role of pericytes in producing EC dysfunction, high levels of glucose may directly lead to EC damage and loss [13, 26, 40].

Retinal fluorescein angiography has revealed areas of nonperfusion in DR [41, 42]. Comparing the results from these studies with retinal digest experiments [43, 44], it is evident that most nonperfused areas in the retina have degenerative pericytes and ECs. These findings may suggest that retinal vascular perfusion is dependent on intact endothelium [45, 46]. Although numerous studies can be cited which demonstrate abnormal platelet function in diabetes, attempts to modify the function have produced much disappointment (reviewed in [47]). Platelets isolated from diabetic animals were shown to be more adherent to the diabetic vasculature than platelets from nondiabetic animals [48]. Thrombocytopenia produced by administering antiplatelet serum caused further retinal blood barrier breakdown [48]. However, assessing other features of DR has revealed no beneficial or deleterious effects of selective antiplatelet drug clopidogrel [49]. These findings indicate that the platelet function may be altered in diabetes; however, the exact role played by the platelets is not clear. More recently, leukocytes were shown to adhere to retinal vasculature [50] and mediate capillary occlusion in streptozotocin-induced diabetic rats [51]. The levels of adhesion molecules (ICAM and VCAM) as assessed by nonquantitative immunohistochemical techniques were shown to be increased or unchanged [52, 53]. Soluble forms of these adhesion molecules have been reported to be higher in the vitreous of patients with PDR [54]. Furthermore, exposure of ECs to either tumor necrosis factor-*α* or advanced glycation end products increases the expression of VCAM [55] providing a mechanistic basis in diabetes.

Sustained hyperglycemia is related to increased vascular permeability [56, 57]. Permeability studies in type 1 and hypertensive type 2 diabetic patients have revealed increased albumin flux through the endothelium [58–65]. The direct cellular and molecular mechanism is still obscure; in part, due to our inability to adequately assess vascular permeability unambiguously. In cultured cells, however, we have shown that glucose- and ET-1 induced EC permeability can be normalized by PKC inhibition [24]. It is believed that a dysfunction in the transfer property of the endothelium occurs, which when precipitated with occlusion, leads to under- or nonperfusion. These areas of nonperfusion, in conjunction with cellular damage, may trigger a reparative response; a finding well documented in retinal microcirculation [40].

### 2.3. Capillary basement membrane (BM) thickening

A consistent feature of DR is thickening of the capillary BMs [66–71]. Capillary BM thickening can result from increased production and decreased degradation of the extracellular matrix (ECM) proteins [67, 69]. High levels of glucose (30 mM) can increase mRNA expression of ECM proteins, collagen and fibronectin (FN), in the kidney mesangial cells and the retinal ECs [72–74]. Retinal BMs of diabetic animals have been shown to contain increased collagen *α*1 (IV), and *β*1 and *γ* chains of laminin, and FN [75]. These changes are brought upon as early as 8 weeks following onset of diabetes [75]. We have also shown that diabetes increases ECM protein expression in the kidney, retina, and heart of streptozotocin-induced diabetic rats [21, 76, 77]. In addition to collagen *α*1 (IV) and FN, upregulation of other ECM proteins such as tenascin has been found in retinal vessels of diabetic patients and animals [78, 79]. However, no difference has been shown in the amount of proteoglycans in BMs of patients with DR [80].

Recently, we have demonstrated that FN undergoes alterative splicing in DR to produce an embryonic isoform ED-B^+^ FN (oncofetal FN) [81]. Increased levels of this isoform are evident in vitreous of patients with PDR and retinal tissues of diabetic rats. This isoform is normally absent in normal adult tissues and may be involved in functional effects via outside-in signaling [22]. In cultured vascular ECs, we have shown that ED-B^+^ FN is involved in VEGF expression and EC proliferation suggesting an important role of this FN isoform in DR.

## 3. MOLECULAR MECHANISMS OF DR PATHOGENESIS

DR is a culmination of numerous biochemical alterations which take place in the vascular tissue of the retina. High levels of glucose affect both the vascular ECs and the pericytes. The retinal ECs incorporate glucose via glucose transporter-1 induced facilitative diffusion [82–84]. Therefore, increased circulating levels of glucose accumulate in the retinal ECs and lead to activation of various biochemical pathways. It is possible that other factors, such as hyperinsulinemia and dyslipidemia, may also contribute to the abnormal signaling in DR. In this review, we will explore the diabetes-induced molecular alterations in the retina and glucose-induced changes in cultured ECs in an attempt to identify novel therapeutic targets for DR.

### 3.1. Aldose reductase (AR) and the polyol pathway

Hyperglycemia-induced secondary complications of diabetes are particularly evident in tissues which are not insulin-sensitive such as the retinal vasculature. Under normal conditions, glucose utilization is primarily through the glycolytic pathway. However, in diabetes excessive glucose is metabolized by the polyol pathway/aldose reductase (AR) enzyme. Increased AR activity is implicated in the development of secondary diabetic complications [85, 86]. Glucose flux through the polyol/AR pathway may lead to sorbitol accumulation [86] and accompanying cellular damage [86, 87] which may be prevented by myo-inositol supplementation [88]. It is believed that increased NADH production, a cofactor for glyceraldehyde 3-phosphate dehydrogenase, leads to augmented levels of glyceraldehyde 3-phosphate. Thus, polyol pathway may also contribute to advanced glycation end product formation through methylglyoxal from increased glyceraldehydes 3-phosphate [89].

Supplementing these experimental evidences of the involvement of AR enzyme in diabetic complications, clinical studies show that polymorphisms in AR gene are linked to increased susceptibility of microvascular complications [90–92]. Clinical trials to assess AR inhibition as a therapeutic modality have not shown any conclusive results. One recent trial with AR inhibitor sorbinil showed slower rate of microaneurysms in the retina [93]. Recently, however, a new class of AR inhibitors was tested in streptozotocin-induced diabetic rats [94]. This selective inhibitor, ARI-809, was as effective in AR inhibition in the animal model as sorbinil. Whether ARI-809 produces more favorable results in clinical trials remains to be determined.

### 3.2. Oxidative stress

**Figure 1:**
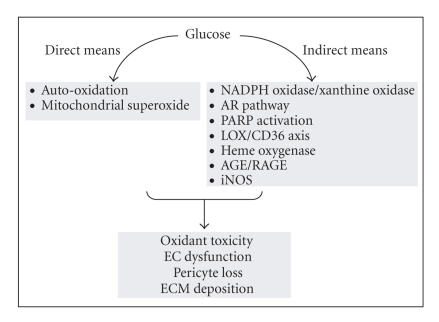
Mechanism and consequence of increased oxidative stress in DR (NADPH, nicotinamide adenine dinucleotide phosphate; AR, aldose reductase; LOX, lipoxygenase; PARP, poly(ADP-ribose) polymerase; AGE, advanced glycation end product; RAGE, receptor for AGE; iNOS, inducible nitric oxide synthase).

Oxidative stress early in the disease state may contribute to EC dysfunction and may also be the driving force in the continued insult to the retinal vasculature during the later stages of the disease. The mechanisms which may underlie increased oxidative stress in ECs and retinal tissues in diabetes are complex and interconnected (Figure 1) [26]. The early pathways of oxidative stress, which may initiate the EC dysfunction and pave the path for continued vascular damage, include glucose auto-oxidation and mitochondrial superoxide production [95–97]. Inhibiting mitochondrial superoxide production has been shown to be beneficial for DR [97].

Oxidative stress in diabetes may also be induced by indirect means, which include the NADPH oxidase enyzme [98, 99]. NADPH oxidase may increase superoxide production and through induction of xanthine oxidase may also inhibit superoxide dismutase. Impairment of antioxidant enzymes could also be carried out by increased AR activity through the imbalance between NADP^+^/NADPH. A number of other enzymes have also emerged as being important mediators of increased oxidative stress. Lipoxygenase enzyme (LOX) may also contribute to diabetes-induced oxidative stress. LOX increases oxidation of low density lipoproteins (ox-LDLs) [100, 101]. We have shown that glucose increases CD36 (an ox-LDL receptor) and leads to increased uptake of ox-LDL and oxidative DNA damage in vascular ECs [100].

Recently, several investigators have shown a role of poly (ADP-ribose) polymerase (PARP) in cultured ECs and retina of diabetic animals [102–104]. Increased PARP activity, possibly in response to oxidative DNA damage, may directly cause vascular EC dysfunction via the depletion of NAD^+^ and ATP. PARP may also cause nuclear factor-*κ*B (NF-*κ*B) activation [105]. In a nondiabetic system, a relationship between PARP activation, histone deacetylases (HDACs) and transcription coactivator p300 has been shown [106, 107]. Whether a similar pathway may also be involved in DR requires further investigation.

### 3.3. Protein glycation

Protein modification has also been shown to play an important role in diabetic complications. Glucose may participate in nonenzymatic glycation of proteins to produce advanced glycation end products (AGEs) [108, 109]. Both AGEs and AGE receptors (termed RAGEs) have been localized to the retinal vasculature and vascular ECs [110–113]. Although the means by which AGE/RAGE cause cellular dysfunction are currently being elucidated. It is believed that AGEs may lead to altered protein function, interfere with the ECM function, and cause elaboration of cytokines. In cultured ECs, AGE formation has been reported to regulate thrombomodulin, ET-1, VEGF, and basic-fibroblast growth factor [114–116]. Administration of exogenous AGEs to diabetic animals show retinal pericyte loss [117]. Retinal ECs, however, proliferate in response to glycated BMs [118]. These studies provide insight into the mechanism of pericyte loss and unregulated EC proliferation in the later stages of the disease. Experimental studies in diabetic animals show that a specific inhibitor of nonenzymatic glycation, aminoguanidine, can prevent retinal microaneurysms, acellular capillaries, and pericyte loss in the diabetic dogs [119].

### 3.4. Protein kinase C (PKC) activation

**Figure 2:**
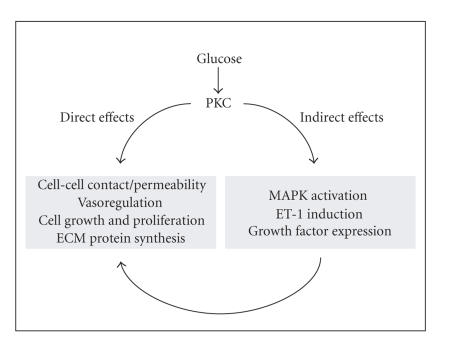
Role of PKC in vascular function in diabetes (DAG, diacylglycerol; PKC, protein kinase C; ECM, extracellular matrix; MAPK, mitogen-activated protein kinase; and ET-1, endothelin-1).

Exposure of cultured ECs to high levels of glucose leads to rapid induction of a number of protein kinase family members [23, 24, 120, 121] suggesting that these proteins may play a role in transducing the adverse effects of hyperglycemia in the retinal vasculature. Several studies have shown the activation of an important protein kinase, PKC [122] in the context of diabetes [23, 24, 122–124]. PKC isoforms which show significant activation in animal models of chronic diabetes include PKC*α*, *β*I, *β*II, *γ*, and *δ* [125, 126]. However, PKC*β*I and II show the highest level of induction in the retina as well as the heart and aorta of the diabetic animals [126]. The mechanism of PKC activation may involve increased diacylglycerol (DAG) levels which have been shown in the retina of diabetic animals [127] and vascular cells exposed to high glucose levels [126, 127]. DAG can be increased through the hydrolysis of phosphatidylinosities or through de novo synthesis. Fatty acid composition analysis of DAG reveal that the major contributor to increased DAG in hyperglycemic conditions may be the de novo synthesis or phospholipase-D mediated production of DAG [127, 128]. PKC activation, either directly or through interaction with other intracellular signaling proteins, may cause a number of vascular effects which are summarized in Figure 2. We and others have previously shown that PKC may mediate glucose-induced EC permeability [24, 129], and ECM protein production [23]. In addition, PKC may be involved in the expression of various growth factors and vasoactive factors, and regulate cellular activity [122, 124, 129–131]. A tremendous amount of work has also been done on the activation of PKC secondary to alteration of growth factors and cytokines. One of the best examples is the activation of PKC through VEGF which is highly relevant to DR. VEGF mediates cellular signaling in ECs through binding to VEGF receptors (VEGF-R1 and VEGF-R2). Upon binding of VEGF, VEGF-Rs signal the release of DAG (through glycolytic pathway intermediates) [132]. This DAG increase causes activation of selective PKC isoforms (predominantly *β* isoform) and mediates the vasoregulatory role of VEGF such as permeability [133]. Invitreal injection of VEGF rapidly increases PKC activation in the retina. Furthermore, the VEGF-mediated retinal permeability can be normalized with PKC-*β* inhibition [133]. We have shown a similar interaction between PKC and ET-1 [24]. Several experimental and clinical studies have been carried out with selective PKC*β* inhibitor, ruboxistaurin mesylate (LY333531) [133–138]. In phase III clinical trials ruboxistaurin showed a delay in the occurrence of moderate visual loss in patients with NPDR at 24 months [139].

## 4. EMERGING TARGETS

### 4.1. Mitogen-activated protein kinase (MAPK)

PKC may also regulate the vascular function in chronic diabetes through interaction with other signaling complexes in the vascular ECs. Recently, studies have reported an important role of MAPK pathway in the diabetic complications [140, 141] which may, in part, be dependent upon PKC activation [23]. MAPK family consists of extracellular signal-regulated kinase (ERK) and stress- activated components, namely c-jun N-terminal kinase (JNK) and p38 (for review, see [140, 142]). MAPK activation proceeds through sequential activation of MAPKKK, MAPKK, and MAPK [142]. We have shown that glucose-induced ECM protein synthesis in ECs may be mediated by the activation of MAPK [23]. We have further demonstrated that such MAPK phosphorylation leads to activation of transcription factors NF-*κ*B and activating protein-1 (AP-1) [23]. Inhibition of either MAPK or PKC is able to normalize the effects of high levels of glucose. Furthermore, inhibiting PKC in cells exposed to high glucose reduces MAPK activation suggesting an important cross-regulation between PKC and MAPK. ECM protein expression through MAPK activation has also been reported in renal tubular cells [143] implicating a role of MAPK in diabetic nephropathy as well [144]. It is, however, possible that MAPK activation may also occur via a PKC-independent pathway [145]. Oxidative stress may cause alternate MAPK activation via ERK5 (big MAPK1/BMK1) [146]. BMK1 knockout results in angiogenic defect and embryonic lethality [147]. BMK1, however, differs from other MAPK as it contains a transcriptional activation domain, mediating protein-protein interaction with several other factors [147, 148]. Whether such pathways are also activated in diabetes remains to be determined.

### 4.2. Activation of other protein kinases

Most recent developments in the attempt to elucidate the signaling events in cultured ECs challenged with high levels of glucose have revealed a role of significantly homologous protein kinase family members, protein kinase B (PKB), [120] and serum- and glucocorticoid-regulated kinase-1 (SGK-1) [121]. Several growth factors stimulate the recruitment of lipid kinases known as Class I phosphoinositide 3-kinases (PI_3_-kinase) to the plasma membrane. These kinases then phosphorylate the glycerophospholipid phosphatidylinositol 4,5-bisphosphate (PtdIns(4,5) P2) converting it to PtdIns(3,4,5)P3 (reviewed in [149, 150]). Evidence indicates that serine/threonine protein kinase PKB may be the key mediator of several downstream events regulated by PI_3_-kinase. There are three major PKB isoforms *α*, *β*, *γ*. These isoforms belong to a subfamily of protein kinases named AGC protein kinases and include PKC and PKA. Following activation of PI_3_-kinase, PKB isoforms are recruited to the plasma membrane and are phosphorylated by phosphoinositide-dependent kinase 1 (PDK-1). PKB can regulate the function of cytoplasmic as well as nuclear proteins [149, 150]. We have demonstrated glucose-induced early activation of PKB [120] and SGK-1 [121]. Inhibiting PKB and SGK-1 either by dominant negative transfections and/or small interfering RNA causes complete normalization of high glucose-induced FN expression in the vascular ECs. This role of PKB in ECM protein expression is regulated by both MAPK and PKC [120]. We have further shown that PKB phosphorylation can lead to the activation of NF-*κ*B and AP-1 [120].

### 4.3. Transcription factors

All extracellular and intracellular signals converge on transcription factors to regulate various aspects of vascular function in diabetes. Two of the major transcription factors which have been shown to be instrumental in mediating the effects of diabetes are NF-*κ*B and AP-1. In nonstimulated cells, NF-*κ*B exists as a latent dimer in the cytoplasm being bound to I*κ*B, an inhibitor protein. Upon stimulation, I*κ*B kinase (IKK) is activated and leads to phosphorylation and subsequent degradation of I*κ*B. Without the inhibitory I*κ*B, NF-*κ*B translocates to nucleus and affects gene expression by binding to *κ*B elements in their promoter region [151]. Oxidative stress is one of the main activators of NF-*κ*B [151]. Interestingly, glucose-induced ET-1 expression may be regulated via NF-*κ*B activation [152]. Studies have indicated nuclear NF-*κ*B immunoreactivity in the pericytes but not retinal vessel ECs of human diabetic eyes [153]. Experimental evidence in diabetic animals, however, shows NF-*κ*B activity in retinal vessel ECs [105, 154–156]. Furthermore, ECs cultured in 25–30 mM glucose also show increased NF-*κ*B activity [104, 105, 135, 157]. Whether the apparent discrepancy is due to the species difference or a result of experimental conditions remains to be determined.

Transcription factor proteins that form the AP-1 dimers are also activated by multiple stimuli mediating several functions [158]. These proteins include the Jun, Fos, and ATF subgroups of transcription factors proteins [158, 159]. Hyperglycemia activates both NF-*κ*B and AP-1 transcription factors via multiple pathways. We have recently shown that MAPK-mediated ECM protein synthesis in ECs is dependent on both NF-*κ*B and AP-1 activation [23]. ET-1 also increases FN expression through the activation of these transcription factors in cultured ECs and target organs of diabetic complications, retina, kidney, and heart [21, 157]. NF-*κ*B is also a redox-sensitive transcription factor which is activated by increased oxidative stress and AGE formation [55, 112, 160]. Several other transcription factors may play regulatory role in these pathways. Recently, a number of clinical trials have shown some promise for the use of triamcinolone acetonide in the management of DR (reviewed in [161]). Triamcinolone acetonide, a synthetic glucocorticoid, has been reported to reduce vascular permeability, hemorrhages, and neovascularization in DR. In addition to regulating VEGF, the mechanisms of action may involve transrepression of both NF-*κ*B and AP-1 [147, 162, 163]. Inactive glucocorticoid receptors are present in the cytoplasm being bound to heat shock protein hsp90 which prevents nuclear translocation. Upon ligand binding (steroid/glucocorticoid), hsp90 dissociates and the receptor/ligand complex translocates to the nucleus. The repression of NF-*κ*B and AP-1 by the glucocorticoid receptors is believed to be due to direct interaction of the receptor with the transcription factors.

### 4.4. Transcription coactivators

Histone-dependent packaging of the genomic DNA is a key mechanism in gene regulation. Remodelling of the chromatin in the nucleus, regulated by acetylation and deacetylation of histone residues, is important in allowing access of transcription factors for DNA binding. Acting in opposing manner, histone acetyl transferases (HATs) and histone decetylases (HDACs) control several cellular processes through transcription factors [164]. p300 and related CREB binding protein (CBP) are the best characterized HATs. p300 associates with CBP associated factor (PCAF) and plays a crucial role in cell differentiation, growth, and apoptosis [164]. Transcription factors such as NF-*κ*B remain inactive, even after nuclear translocation, without association with p300 [164, 165]. NF-*κ*B activity in diabetes is regulated by p300. We have shown that FN expression, in both cultured ECs and in the retina of diabetic rats, is mediated by p300 induction [104]. It is not clear yet whether HDACs may modulate such pathways. This signaling pathway is modifiable through protein kinase C, protein kinase B, and possible MAPK [104]. These results suggest that diabetes-induced molecular alterations, oxidative stress, PKC and MAPK activation may converge on NF-*κ*B to mediate increased ECM protein expression in the vascular tissue.

## 5. CONCLUDING REMARKS

**Figure 3:**
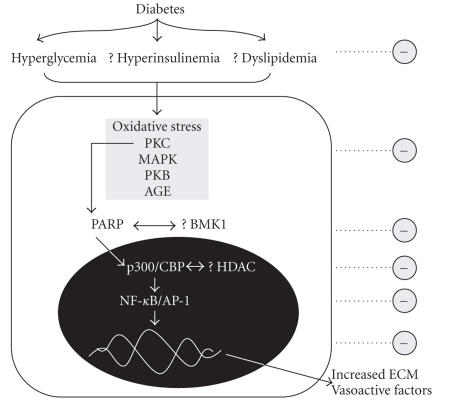
Putative signaling mechanisms in vascular ECs and possible treatment targets for DR (? = hypothetical; ⊖ = potential therapeutic target, PARP = poly(ADP-ribose) polymerase; AGE = advanced glycation end product, PKC = protein kinase C; PKB = protein kinase B, ECM = extracellular matrix; MAPK = mitogen-activated protein kinase, Ox = oxidative stress, BMK1 = big MAPK1, HDAC = histone deacetylase).

Diabetes leads to selective damage to a number of target organs including the retina. Studies have identified common signaling pathways in cultured cells and animal models of diabetes which may in concert lead to the pathogenetic changes in the retinal vasculature. One recurring theme in DR and other complications of diabetes is vascular EC dysfunction. This endotheliopathy is carried out by hyperglycemia-induced changes in vasoregulation (ET-1/NO), oxidative stress, and increased polyol pathway. Altered EC function then is precipitated with continued activation of intracellular signaling proteins such as PKC, PKB, and MAPK, finally culminating in pathological induction of transcription factors such as NF-*κ*B and AP-1. HATs such as p300 may represent upstream regulators of these transcription factors. The concept of such epigenetic regulation of gene transcription is intriguing. One integral question in adequate combat of the secondary complications of diabetes is how does one inhibit such a complex and interrelated signaling cascade? A possible avenue is to identify a point of convergence which may reside at the epigenetic level. The activity (including the specificity) of transcription factors is also dependent on the coactivators in the protein complex. A further grasp of the hyperglycemia-induced transcription machinery (transcription profiling) is crucial to identifying the molecular signature of DR. Future advances in EC biology would greatly enhance our ability to simultaneously inhibit these signaling proteins, and prevent and possibly reverse the adverse effects of chronic diabetes. An outline of the discussed signaling mechanisms and potential treatment targets is shown in Figure 3.
